# USING HOSPITAL ELECTRONIC HEALTH RECORDS TO DETERMINE FRAILTY WITH THE PICTORIAL FIT-FRAIL SCALE

**DOI:** 10.1093/geroni/igad104.2799

**Published:** 2023-12-21

**Authors:** Alanna Bohnsack, Karla Faig, Allyson Cook, Cameron MacLellan, Susan Benjamin, Josh Shanks, Sherry Gionet, Pamela Jarrett

**Affiliations:** Horizon Health Network, Saint John, New Brunswick, Canada; Horizon Health Network, Saint John, New Brunswick, Canada; Dalhousie University, Saint John, New Brunswick, Canada; Dalhousie University, Saint John, New Brunswick, Canada; Trauma New Brunswick, Saint John, New Brunswick, Canada; Dalhousie University, Saint John, New Brunswick, Canada; Horizon Health Network, Saint John, New Brunswick, Canada; Horizon Health Network, Saint John, New Brunswick, Canada

## Abstract

Hip fractures are common in older adults, particularly those that are frail. There is no “gold standard” tool to measure frailty retrospectively from hospital Electronic Health Records (EHRs). The aim of this research was to explore the utility of the Pictorial Fit-Frail Scale (PFFS) in determining frailty retrospectively in older adults admitted to hospital with a hip fracture. A random sample of 200 patients who had a hip fracture was extracted from a larger sample of 682 hip fractures that occurred in older adults (65 years and older) admitted to a Level 1 Trauma Center (April 2015-March 2019). Each EHR was reviewed to determine the availability of data and the score for each of the 14 PFFS domains. The majority, 189 (94.5%) of the EHRs, had data to complete 11-14 of the domains. Patient information was often unavailable to complete the domains of daytime tiredness and pain. The average age was 83.2 (SD 8.2), and 73.0% were female. The mean Raw PFFS score prior to admission was 9.7 (SD 6.6), and 45.5% of patients were classified as moderately or severely frail. Using the Standardized PFFS score (adjusted for up to three missing domains), the mean score was 11.8 (SD 7.9), and 58.2% of patients were classified as moderately or severely frail. The hospital EHR can be used to complete the PFFS. This finding provides researchers and healthcare professionals with a way to stratify older hospitalized patients by frailty, which may be a useful tool for evaluating health outcomes.

